# Exogenous auxin represses soybean seed germination through decreasing the gibberellin/abscisic acid (GA/ABA) ratio

**DOI:** 10.1038/s41598-017-13093-w

**Published:** 2017-10-03

**Authors:** Haiwei Shuai, Yongjie Meng, Xiaofeng Luo, Feng Chen, Wenguan Zhou, Yujia Dai, Ying Qi, Junbo Du, Feng Yang, Jiang Liu, Wenyu Yang, Kai Shu

**Affiliations:** Key Laboratory of Crop Ecophysiology and Farming System in Southwest China (Ministry of Agriculture), Sichuan Engineering Research Center for Crop Strip Intercropping System, Institute of Ecological Agriculture, Sichuan Agricultural University, Chengdu, 611130 China

## Abstract

Auxin is an important phytohormone which mediates diverse development processes in plants. Published research has demonstrated that auxin induces seed dormancy. However, the precise mechanisms underlying the effect of auxin on seed germination need further investigation, especially the relationship between auxins and both abscisic acid (ABA) and gibberellins (GAs), the latter two phytohormones being the key regulators of seed germination. Here we report that exogenous auxin treatment represses soybean seed germination by enhancing ABA biosynthesis, while impairing GA biogenesis, and finally decreasing GA_1_/ABA and GA_4_/ABA ratios. Microscope observation showed that auxin treatment delayed rupture of the soybean seed coat and radicle protrusion. qPCR assay revealed that transcription of the genes involved in ABA biosynthetic pathway was up-regulated by application of auxin, while expression of genes involved in GA biosynthetic pathway was down-regulated. Accordingly, further phytohormone quantification shows that auxin significantly increased ABA content, whereas the active GA_1_ and GA_4_ levels were decreased, resulting insignificant decreases in the ratiosGA_1_/ABA and GA_4_/ABA.Consistent with this, ABA biosynthesis inhibitor fluridone reversed the delayed-germination phenotype associated with auxin treatment, while paclobutrazol, a GA biosynthesis inhibitor, inhibited soybean seed germination. Altogether, exogenous auxin represses soybean seed germination by mediating ABA and GA biosynthesis.

## Introduction

The legume species soybean (*Glycine max* L.) is one of the most important oil crops in the world, and is an important source of plant oil and protein for human consumption^1^. For soybean, timely germination and uniform seedling emergence from the soil under natural field conditions are crucial to high yield. On one hand, the seeds of soybean possess high oil and protein concentrations, compared to cereal crops such as wheat and rice. Subsequently, the oxidation of oil and protein causes seeds to deteriorate during storage, resulting in decreased soybean seed germination and seedling emergence in the field^2,3^. On the other hand, the pre-harvest sprouting of soybean seeds also causes significant reductions in soybean yield and quality, especially under conditions of high temperature and humidity^2,4^. Combined with the rigid and impermeable seed coat of soybean^5^, these limitations constrain soybean seed quality, and can negatively affect soybean seed germination. Consequently, further investigations of the precise physiological and molecular mechanisms underlying soybean seed germination is of both applied and fundamental relevance.

Seed germination is a key stage during a plant’s life-cycle, and the germination process is determined by diverse environmental cues, such as the availability of suitable levels of light, water and oxygen, as well as the presence of endogenous phytohomones^4,6,7^. Abscisic acid (ABA) promotes seed dormancy and thus inhibits seed germination, while gibberellins (GAs) release seed dormancy and promote seed germination. These are the key hormonal regulators of seed dormancy and germination, and have been very well studied over past decades^6,8–10^. Mutants altered with respect to ABA and/or GA biosynthesis or signaling pathways always show an altered germination phenotype. In the model plant *Arabidopsis*, for instance, the ABA biosynthesis mutants *nced3*, *nced5*, *nced6* and *aba2* ^11,12^, and the ABA signaling mutants *abi3* ^13^, *abi4* ^14^, and *abi5* ^15^ exhibited a faster-germinating phenotype compared to the wild-type, whereas mutants overexpressing either *ABA2* or *ABI4* exhibited a deep level of dormancy, resulting in the delayed-germination phenotype^14,16^.

In contrast to ABA mutants, seeds of the GA-deficiency mutants, *ga1* and *ga2*, failed to germinate unless exogenous GA treatment was applied^14,17^, while mutants defective in GA 2-oxidases (GA2ox), enzymes which deactivate bioactive GA, showed decreased seed dormancy levels and faster-germination phenotypes^18^. Further, the ratios including GA_1_/ABA and GA_4_/ABA is the key determinant of seed germination^4,6^. Altogether, both ABA and GA signaling/level play key roles in the regulation of seed germination.

Another important phytohormone, auxin, is involved in almost all stages of plant development, including root growth, apical dominance, fruit growth and response to environmental signals^19–24^. Auxin also interacts with ABA to regulate plant drought and osmotic stress responses in Chinese kale (*Brassica oleracea* var. *alboglabra*)^25^. In addition, ABA and auxin signaling are also mediated by the type B Gγ subunit SlGGB1 in tomato, with silencing of *SlGGB1* affecting seed development, lateral root growth and fruit shape formation^26^. Recently, auxin was demonstrated to be a regulator of several phytohormone signaling pathways, including ABA and GA, and, as a result, regulated tomato fruit formation^27^.

Regarding seed germination processes, earlier studies showed that exogenous application of the auxin indole-3-acetic acid (IAA) delayed wheat seed germination and inhibited pre-harvest sprouting on mother plants^28^, while exogenous auxin treatment also inhibited seed germination under salt stress conditions in *Arabidopsis* ^29^, although the mechanisms underlying the inhibitory effects of auxin on seed germination have still to be elucidated. A recent study revealed that auxin induced seed dormancy through enhancing ABA signal transduction, identifying auxin as a promoter of seed dormancy^30^. Detailed investigation showed that *ABI3* is required for auxin-activated seed dormancy. The auxin-responsive transcription factors, *ARF10* and *ARF16*, indirectly promote *ABI3* transcription, and consequently maintain seed dormancy levels and repress germination^30^. However, there are some important scientific questions which remain unanswered. For instance, does auxin regulate ABA biosynthesis during seed germination, and, if so, how does that happen? Furthermore, the relationship between auxin and GA biosynthesis is still not fully clear. Therefore, further investigations are needed to identify the missing links in the impact of auxin on the ABA and GA biosynthesis pathways and signal transduction cascades.

In this study, seeds of the important oil crop soybean were employed as experimental material. The objectives were firstly to identify the effect of exogenous auxin treatment on soybean seed germination, and to determine the effects of fluridone (FL), an ABA-biosynthesis inhibitor, and paclobutrazol (PAC), a GA-biosynthesis inhibitor, on the auxin-induced delayed-germination phenotype. Secondly, the mode of action of the exogenous auxin effect on germination was to be analyzed, using quantitative real-time PCR (qPCR) to quantify the transcriptional level of key genes of the ABA and GA biosynthetic pathways, Gene expression data were complemented with phytohormone quantification data during the process of seed germination in the presence or absence of exogenous auxin, in order to identify changes in key GA/ABA ratios associated with particular altered-germination phenotypes. The ultimate objective was to determine how exogenous auxin treatment affected germination in terms of response to ABA and/or GAs.

## Results

### Exogenous auxin negatively regulates soybean seed germination

**Figure 1:**
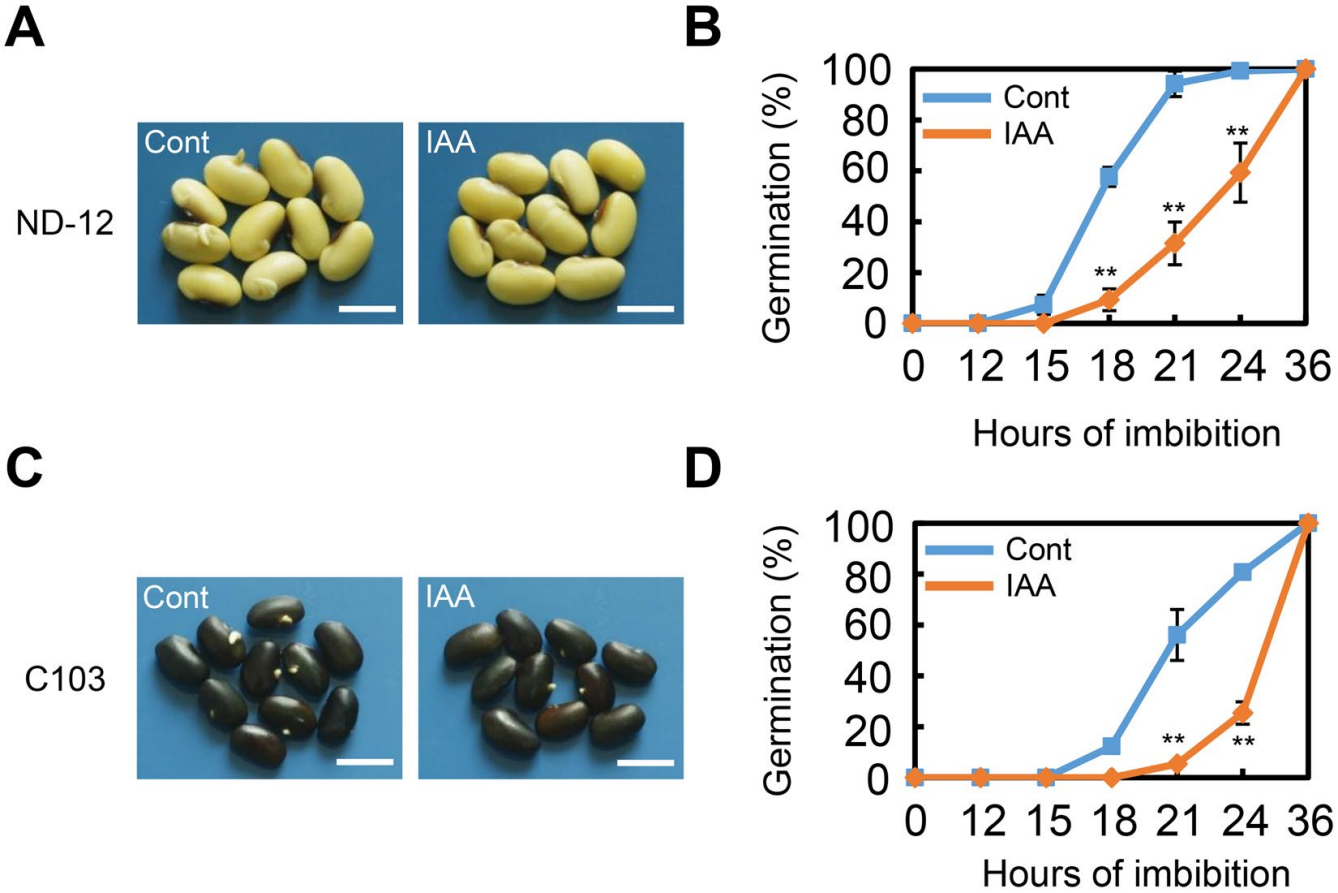
Exogenous IAA treatment represses soybean seed germination under dark conditions. Healthy and elite soybean seeds (cultivars ND-12 and C-103) were incubated on two layers of filter paper in Petri dishes. The concentration of IAA used was 1 μM, and the equivalent amount of ultrapure water was added as control (Cont). The germination rates under dark conditions were recorded using a safe green light. Quantitative analysis of germination rates is shown in the right panels. The representative images (21 hours after sowing) are shown (left panels). (**A**,**B**) for cultivar ND-12; (**C**,**D**) for cultivar C-103. Bar in panel A and C = 10 mm. The average percentages of four repeats ± standard error are shown. **Difference is significant at the 0.01 level.

Indole-3-acetic acid (IAA) is the most common and naturally-occurring of the auxins and has been frequently employed in the auxin-related research field^31^. Initially, we investigated the effect of exogenous auxin treatment on seed germination of the soybean cultivar ND-12 (Nandou-12). The results showed that exogenous IAA treatment significantly delayed the seed germination process (Fig. 1A). The speed of germination of IAA-treated soybean seeds was two- to three- fold slower than that of the control (“Cont”) between 15 and 36 h of the imbibition time course (Fig. 1B). To confirm the inhibitory effect of exogenous auxin treatment on soybean seed germination, another cultivar, C-103, which is distinct from ‘ND-12’ in terms of genetic background, was tested. Similar to the effect on ‘ND-12’, IAA treatment also markedly delayed ‘C-103’ seed germination (Fig. 1C,D).

**Figure 2:**
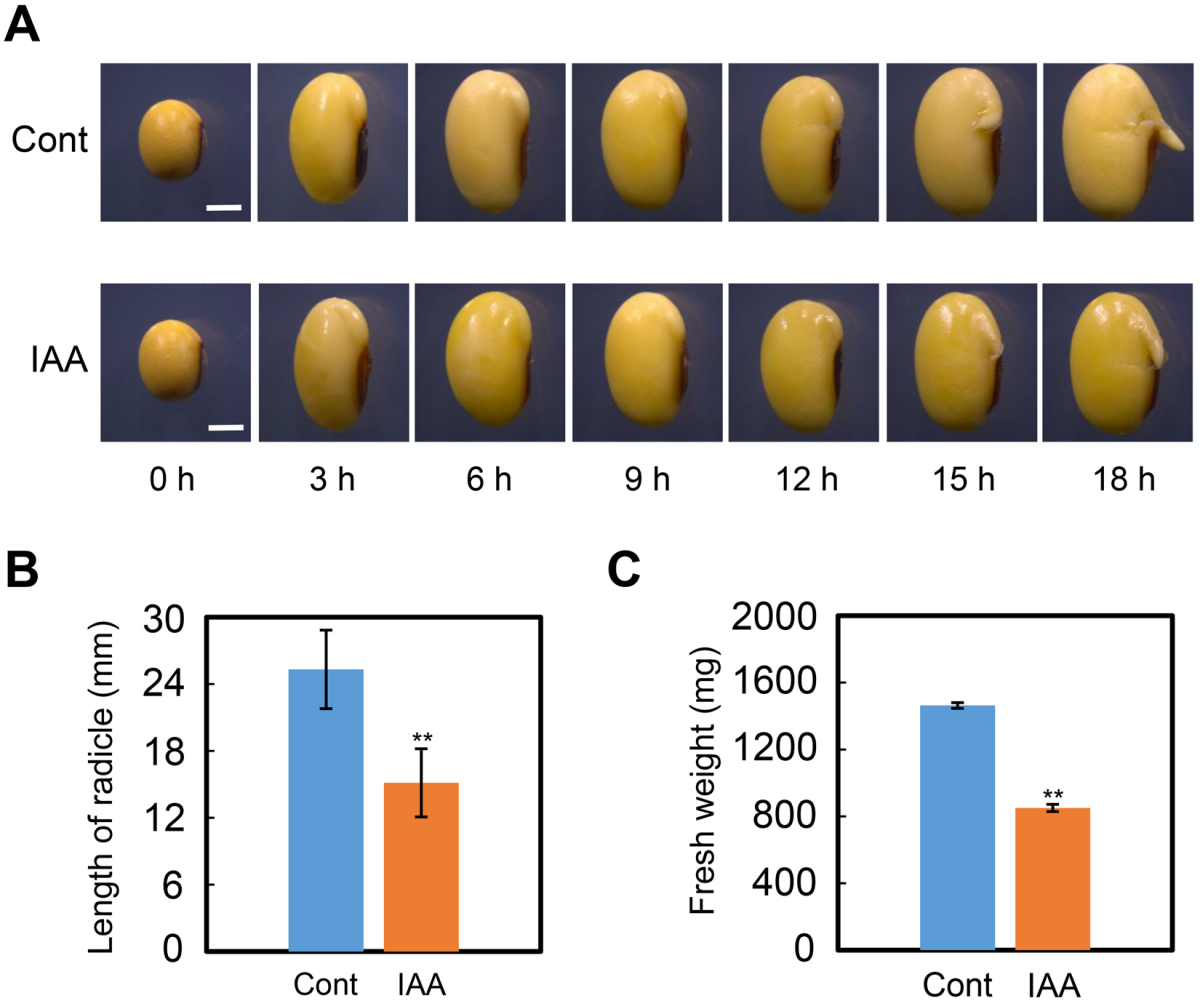
IAA delay soybean seed coat rupture and radicle protrusion. The imbibition seeds treated by IAA (1 μM) and H_2_O were investigated by stereomicroscope (Leica M165 C, Germany) with different time points (0, 3, 6, 9, 12, 15, 18 hour after sowing). (**A**) The representative photographs of seed coat rupture of IAA and H_2_O treatment soybean seeds during imbibition. (**B**) Length of radicle of germinated soybean seeds 48 hours after IAA treatment. (**C**) Fresh weight of germinated soybean seeds 48 hours after IAA treatment. Bar in panel A = 50 mm. The average percentages of four repeats ± standard error are shown. **Difference is significant at the 0.01 level.

The earliest visible stages of seed germination involve the processes of seed coat rupture and radicle protrusion^6,10,15^. The effect of IAA treatment on those processes was investigated by microscope observation. The results revealed that, in ‘ND-12’, seed coat rupture and radicle protrusion were delayed by exogenous IAA treatment (Fig. 2A). At 15 hours after sowing, the control, treated with H_2_O, showed normal coat rupture and protrusion of the radicle, while both processes were markedly delayed in IAA-treated seed (Fig. 2A). The effect of exogenous IAA treatment on post-germination seedling growth was then analyzed. The data showed that the radicle length of germinated seeds after IAA treatment was significantly shorter than that of H_2_O-treated seeds (Fig. 2B), while a significant inhibitory effect of IAA on the fresh weight of germinated seeds was also detected (Fig. 2C).

The inhibitory effect of IAA on seed germination in ‘ND-12’ was exhibited in a concentration-dependent manner, with the more obvious delay effect being detected at higher concentrations (Supplementary Fig. S1A, S1B). In addition, the post-germination growth parameters, including radicle length (Supplementary Fig. S1C) and seedling fresh weight (Supplementary Fig. S1D), showed similar dosage-response curves to exogenous IAA treatment as did germination.

Given that a range of synthetic auxins with different structures are available, such as α-naphthalene acetic acid (NAA) and 2,4-dichlorophenoxyacetic acid (2,4-D), these auxins were also tested in this research. The results revealed effects similar to that of IAA. NAA and 2,4-D also exhibited the delaying effect on soybean seed germination, with 2,4-D showing an effect greater than that of NAA at the same concentration (Supplementary Fig. S2A, S2B). We concluded that exogenous auxin treatment negatively regulates the soybean seed germination process through delaying seed coat rupture, and repressing radicle protrusion.

### Auxin enhances ABA biosynthesis but represses GA biosynthesis

The aim of the next element of the research was to identify the missing links between the inhibitory effect of IAA on soybean seed germination, and the interactions between auxin and both GA and ABA, which are the primary regulators of the seed germination processes^4,10^. In a previous study, ABA exhibited the classical inhibitory effect on soybean seed germination, while GA promoted seed germination^32^. To analyze the effect of auxin on the ABA-/GA-regulated process of germination, the transcription patterns of genes involved in ABA/GA biosynthesis and signaling were investigated in a time-course analysis in dry and imbibed soybean seeds by qPCR, with *GmACT11* being used as the reference gene^33–35^.

**Figure 3:**
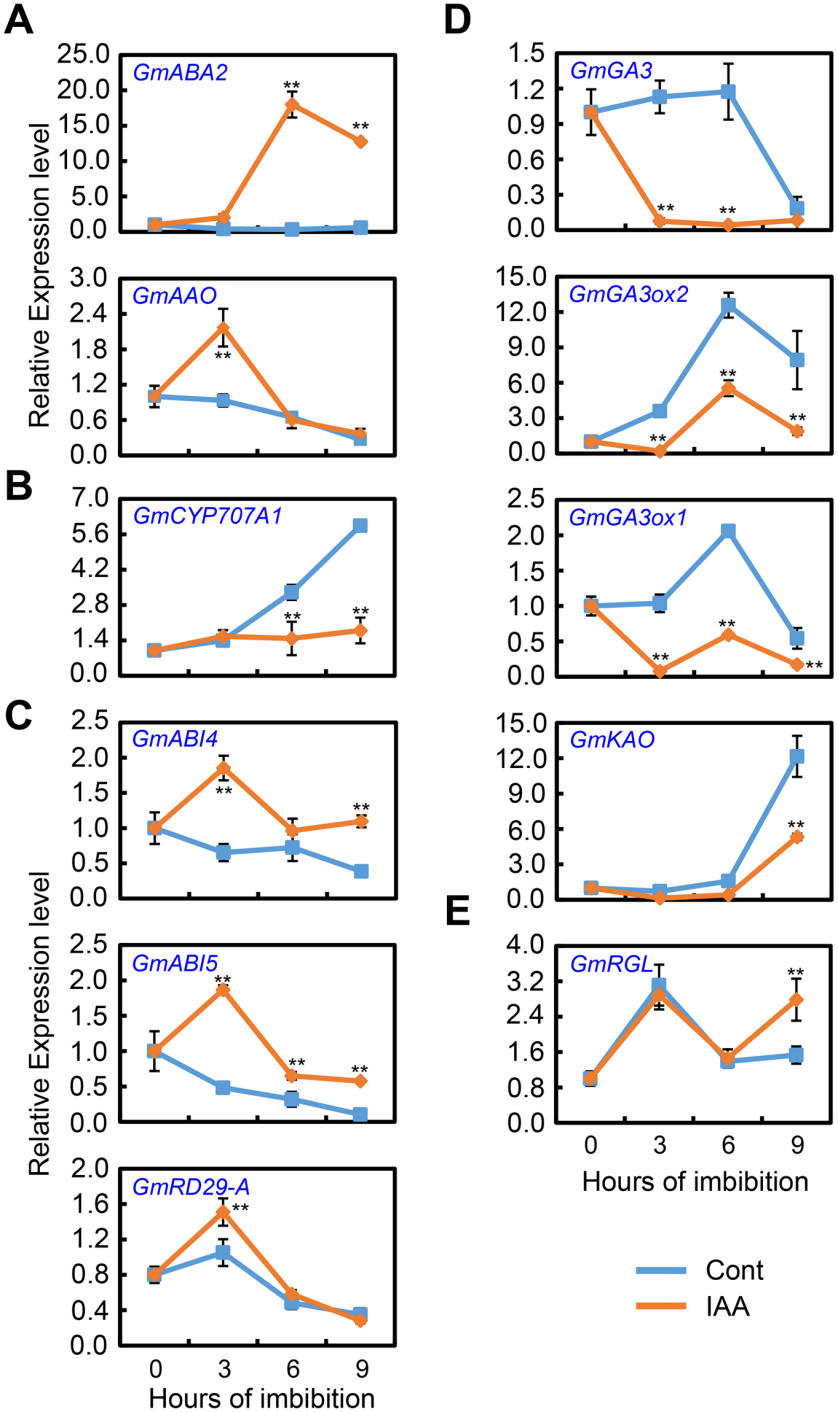
Gene transcription pattern analysis during the course of seed imbibition after IAA treatment (1 μM). Gene expression was investigated by qRT-PCR assay. Dry seeds and imbibed seeds (0, 3, 6, 9 hours after sowing) were used for total RNA isolation. Three biological replications were performed. (**A**) ABA biosynthesis gene, *GmABA2* and *GmAAO*. (**B**) ABA inactivation gene, *GmCYP707A1*. (**C**) Positive regulators genes of ABA signaling pathway, *GmABI4*, *GmABI5* and *GmRD-29A*. (**D**) GA biosynthesis genes, *GmGA3*, *GmGA3ox2*, *GmGA3ox1*and *GmKAO*. (**E**) Negative regulators genes of GA signaling pathway, *GmRGL*. *GmACT11* was used as reference gene for qRT-PCR. **Difference is significant at the 0.01 level.

The qPCR analysis data revealed that, compared to the control, the transcript levels of the soybean ABA biosynthesis genes *GmABA2* and *GmAAO* were significantly up-regulated by IAA treatment during seed imbibition (Fig. 3A). For *GmABA2*, the higher transcription level was maintained throughout the imbibition process, while *GmAAO* showed the elevated expression level at 3 hours after sowing (Fig. 3A). Given that ABA content is regulated by the balance between the anabolic and catabolic pathways^36^, the expression patterns of the ABA-inactivation genes were also investigated. The result showed that the ABA catabolism gene *GmCYP707A1* exhibited a significantly lower transcription level than the control after IAA treatment (Fig. 3B). Given the enhanced transcription of the ABA biosynthesis genes and the decreased expression of the ABA catabolism gene, we next investigated the transcription levels of genes involved in the ABA signaling pathway. As shown in Fig. 3C, exogenous IAA treatment markedly enhanced ABA signaling by up-regulating *GmABI4*, *GmABI5* and *GmRD29-A* transcription, in which *GmABI4* and *GmABI5* are the key positive regulators in the ABA signaling pathway, while *GmRD29-A* is a downstream ABA-response gene^6^. Altogether, transcription analysis demonstrated that ABA biosynthesis and signaling were induced by exogenous IAA treatment during the seed germination process in soybean.

ABA and GA regulate the seed germination process antagonistically, and both phytohormones also suppress biosynthesis of the other^11,37^. Therefore, the effect of IAA on the transcription of key genes involving in the GA biosynthesis and signaling pathways during soybean seed germination was examined. The qPCR analysis revealed that the expression of *GmGA3*, *GmGA3ox2*, *GmGA3ox1* and *GmKAO* decreased significantly after IAA treatment (Fig. 3D). The transcription level of *GmGA3* of IAA-treated seeds was significantly lower than that of the control, and this pattern was maintained from 3 hours after sowing. *GmGA3ox2*, *GmGA3ox1* and *GmKAO* showed the similar expression pattern between IAA treatment and the control, and the decreased pattern was also detected (Fig. 3D). The transcription of *GmRGL*, which encodes the DELLA transcription regulator protein, which represses seed coat rupture during the *Arabidopsis* seed germination process^38^, was up-regulated slightly during imbibition in the presence of IAA (Fig. 3E). Taken together, these analyses revealed that exogenous IAA treatment appeared to negatively regulate GA biosynthesis and signaling during soybean seed germination.

**Figure 4:**
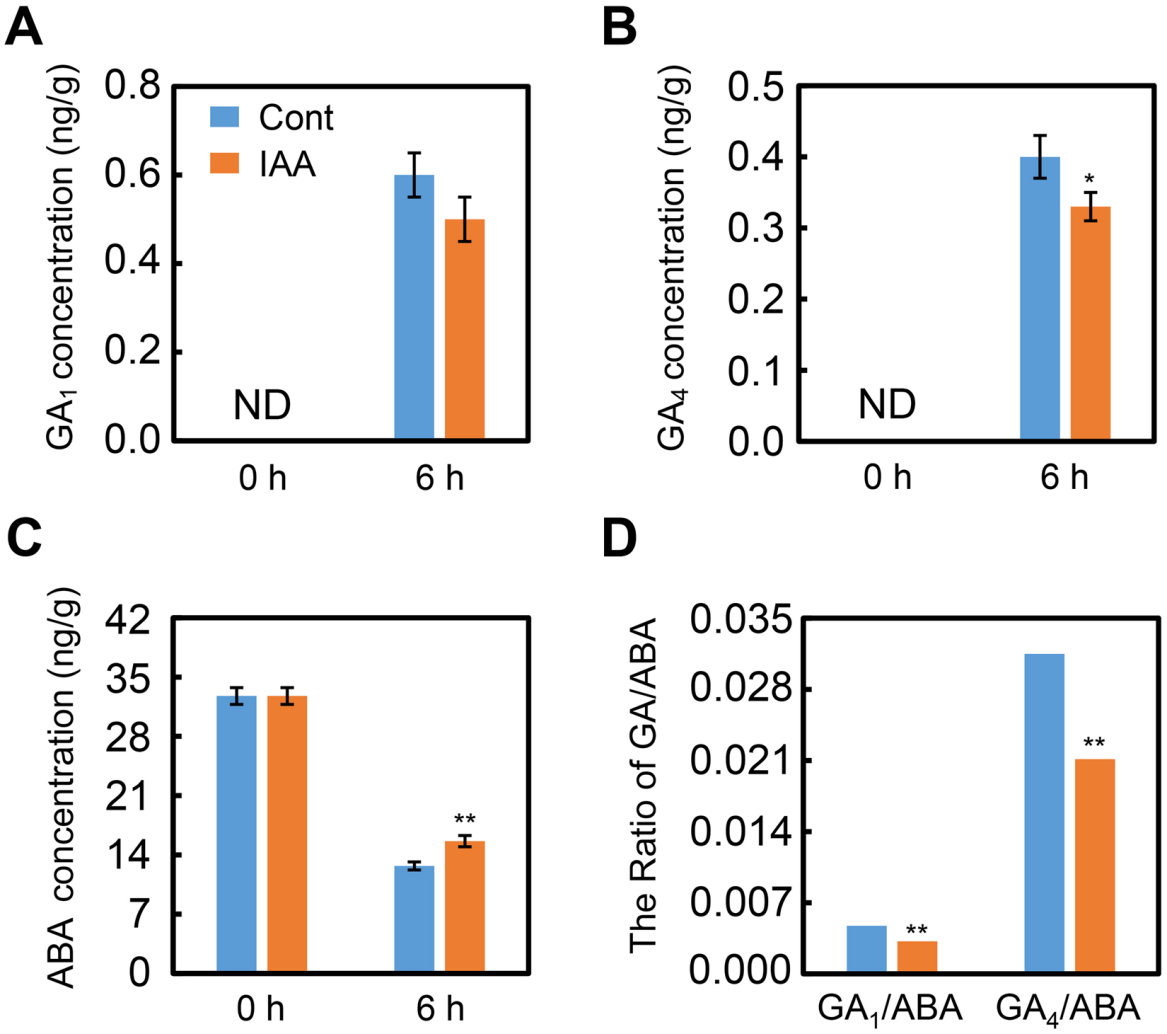
IAA application decreases the ratio between GA_4_ and ABA. Soybean seeds were incubated under dark condition at 25 °C and treated with 1 μM IAA. Equivalent ultrapure water was added as control (Cont). Dry seeds and 6-h imbibed seeds were used to determine the concentration of endogenous active GA_1_ (**A**), active GA_4_ (**B**) and ABA (**C**). The ratios including GA_1_/ABA and GA_4_/ABA are also shown (**D**). *Difference is significant at the 0.05 level, while **difference is significant at the 0.01 level.

The qPCR data indicated that IAA appears to mediate ABA and GA biosynthesis through regulating the expression of the respective biosynthetic genes, signaling genes and catabolic genes. To test this hypothesis, the concentrations of endogenous ABA and active GAs in imbibed soybean seeds were quantified. The results revealed that IAA treatment decreased active GA_1_ and GA_4_ levels (Fig. 4A,B), resulting, in particular, in a marked decrease in GA_4_ content (Fig. 4B). In contrast, ABA concentration in IAA-treated soybean seed during imbibition increased significantly (Fig. 4C). Consequently, the ratios GA_1_/ABA and GA_4_/ABA were down-regulated (Fig. 4D); the significance of this effect is that the GA/ABA ratio is a key factor for seed germination^6,10,37^. Consequently, the concentrations of both ABA and GAs was in line with both the gene expression analysis and the phenotype descriptions. Altogether, exogenous IAA treatment positively regulated ABA concentration while negatively mediating GA concentration.

### FL reverses the delayed-germination phenotype of auxin-treated seeds

To further confirm the observation that exogenous auxin treatment enhances ABA biosynthesis while repressing GA biosynthesis during the soybean seed germination process, the response of soybean seed germination to FL, an ABA biosynthesis inhibitor, and to PAC, a GA biosynthesis inhibitor, during imbibition was investigated.

**Figure 5:**
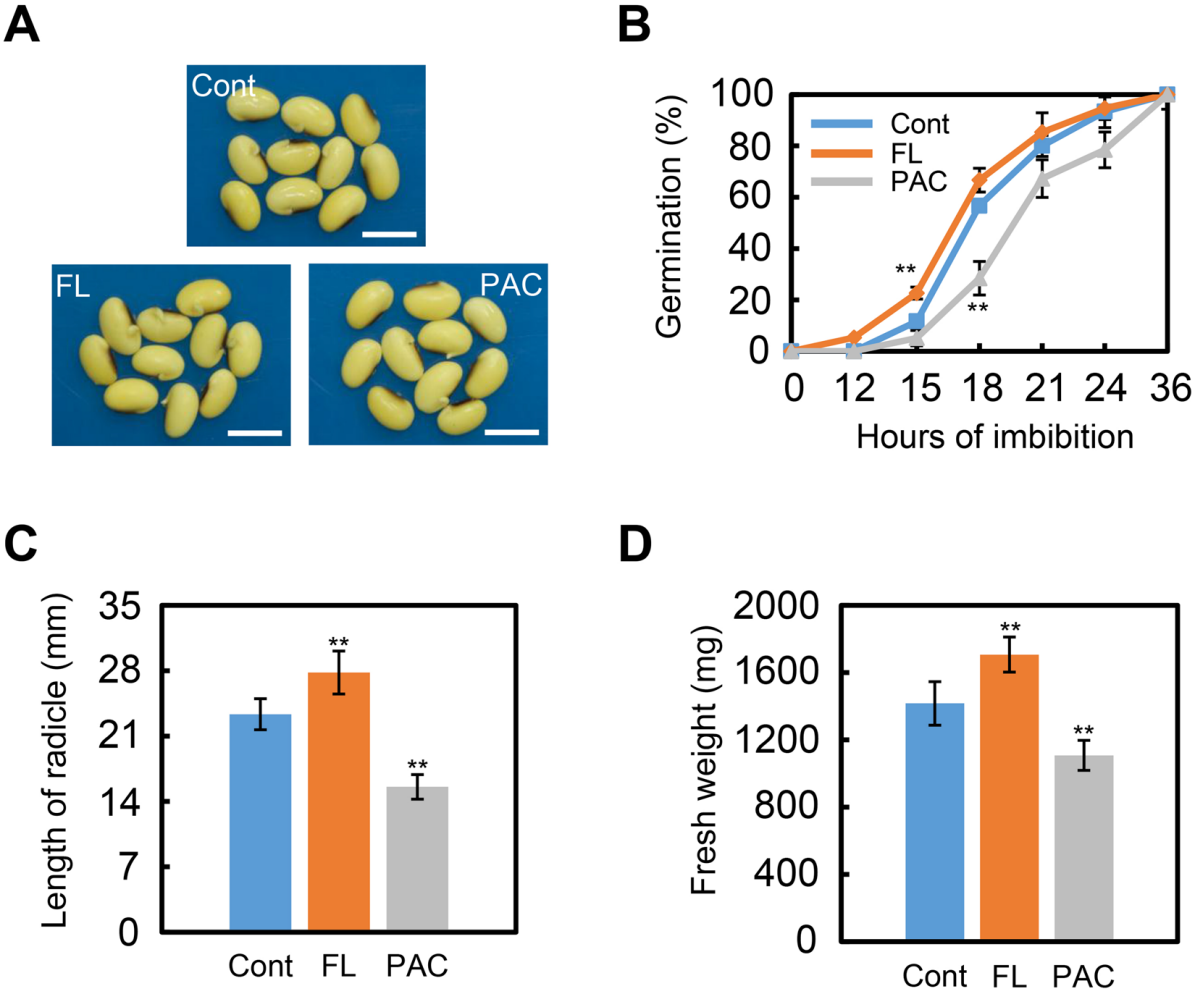
The responsiveness of soybean seeds to ABA biogenesis inhibitor FL and GA biogenesis inhibitor PAC. Cultivar ND-12 was employed in this experiment. (**A**) The representative photographs during seed germination (21 hours after sowing) are shown. (**B**) Quantitative analysis of germination rates are shown. (**C**) Length of radicle of germinated soybean seeds after FL or PAC treatment (48 hours after sowing). (**D**) Fresh weight of germinated soybean seeds after FL or PAC treatment (48 hours after sowing). **Difference is significant at the 0.01 level. Bar in panel A = 10 mm.

Using ‘ND-12’ as the cultivar, the data revealed that FL promoted seed germination, while PAC delayed this process (Fig. 5A,B). The response of post-germination growth parameters, such as radicle length (Fig. 5C) and seedling fresh weight (Fig. 5D), paralleled these germination data. When the experiment was repeated with ‘C-103’ seeds, similar conclusions were arrived at (Supplementary Fig. S3). Germination of ‘C-103’ seeds was promoted by FL treatment, while exogenous PAC markedly delayed C-103 seed germination (Supplementary Fig. S3A, S3B), while the radicle length (Supplementary Fig. S3C) and seedling fresh weight (Supplementary Fig. S3D) responses to FL and PAC resembled those from cultivar ND-12.

**Figure 6:**
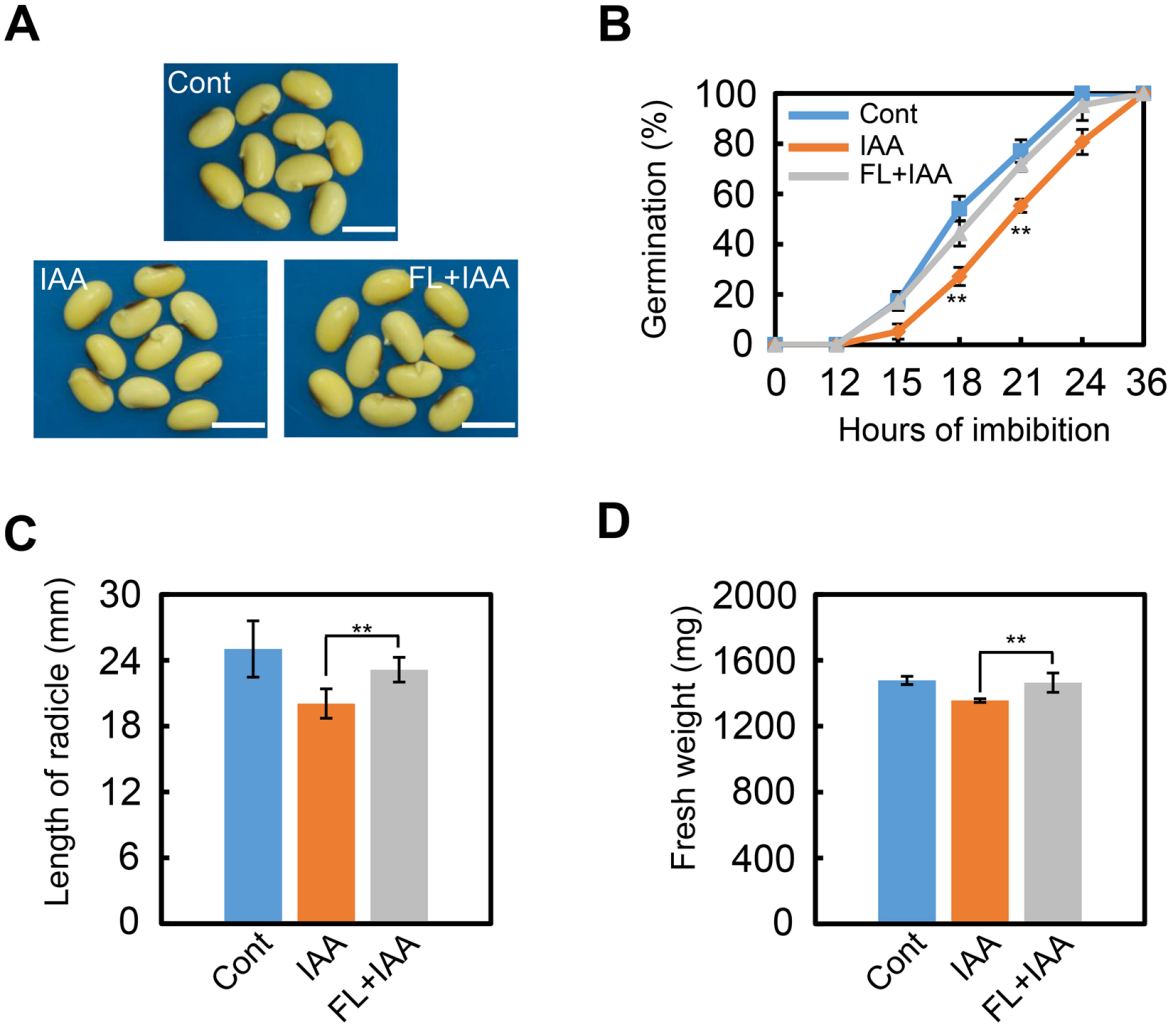
FL rescues the delayed-germination phenotype after IAA treatment. Cultivar ND-12 was employed in this experiment. Soybean seeds were incubated on two layers of filter paper in Petri dishes at 25 °C and treated with 1 μM IAA and 100 nM FL + 1 μM IAA, respectively. Cont, IAA, IAA + FL three treatments were shown. (**A**) The representative photographs during seed germination (21 hours after sowing) are shown. (**B**) Quantitative analysis of germination rates are shown. (**C**) Length of radicle of germinated soybean seeds after IAA or IAA + FL treatments (48 hours after sowing). (**D**) Fresh weight of germinated soybean seeds after IAA or IAA + FL treatments (48 hours after sowing). **Difference is significant at the 0.01 level. Bar in panel A = 10 mm.

As described previously, IAA treatment promotes ABA biosynthesis while repressing ABA catabolism (Figs 3 and 4). It is known that FL blocks the ABA biosynthesis pathway, so it was hypothesized that that FL could reverse the delayed-germination phenotype of IAA-treated seeds resulting from the promotion of ABA biosynthesis. The data showed that FL indeed restored the normal-germination phenotype of IAA-treated ‘ND-12’ seeds (Fig. 6A,B), while the post-germination parameters such as radicle length (Fig. 6C) and fresh weight (Fig. 6D) responded similarly, further supporting these conclusions. Altogether, these investigations demonstrated that IAA indeed promoted ABA biosynthesis during the soybean seed germination process.

## Discussion

Although auxin has been shown to interact with ABA and GA^39^, auxin alone has not been considered to be a key regulator of the seed germination process. Earlier studies revealed that exogenous auxin application significantly suppressed *Arabidopsis* seed germination under salt stress conditions^29^, indicating that auxin plays an important role in seed germination in response to environmental stimuli. It has also been reported that IAA could inhibit pre-harvest sprouting during wheat seed maturation^28^, while ABA repressed embryonic axis elongation by enhancing auxin signaling during seed germination^40^. However, the precise mechanisms underlying the effects of auxin on seed germination remained largely unknown until very recently.

Emerging genetic evidence has demonstrated that auxin promotes and regulates seed dormancy, alongside the ABA signaling pathway. ARF10 and ARF16, the auxin-responsive transcription factors, can indirectly activate *ABI3* transcription, to inhibit the seed germination process^30^. Furthermore, another research group also demonstrated that seeds of the *Arabidopsis abi4* and *abi5* mutants are insensitive to auxin treatment during germination, indicating that ABI4 and ABI5 are important regulators of auxin-mediated inhibition of seed germination^41,42^. Recently, *GSR1* (*Germostatin Resistance Locus 1*), encoding a tandem plant homeodomain (PHD) finger protein, was dissected^43^. Further biochemical, genetic and phenotypic analyses revealed that GSR1 forms a co-repressor with ARF16 to regulate seed germination^43^, indicating that GSR1 may be a member of an auxin-mediated seed germination genetic network. These studies demonstrated that auxin regulates seed germination primarily through mediating the ABA signaling pathway, but it was still unclear as to how auxin interacts with the ABA biosynthesis and GA biosynthesis/signaling pathways in the seed germination regulatory network.

In this study, the phenotypic description, gene transcription pattern analysis and phytohormone measurement demonstrated that auxin indeed promotes ABA biosynthesis while repressing GA biosynthesis, and it also mediates the ABA- and GA- signaling transduction pathways. Treatment with different auxins, including IAA, NAA and 2, 4-D, showed similar inhibitory effects on soybean seed germination, while cultivars with different genetic backgrounds responded similarly to exogenous auxin treatment (Figs 1, 2 and S2). In addition, the repression effect of IAA on soybean seed germination showed a concentration-dependent response (Fig. S1). Subsequently, qPCR analysis revealed that IAA treatment significantly enhanced ABA-biosynthesis gene expression while inhibiting transcription of an ABA-inactivation gene (Fig. 3A,B). ABA signaling was promoted by exogenous IAA treatment (Fig. 3C). IAA also negatively regulated transcription of GA-biosynthesis genes, and repressed GA-signaling transduction (Fig. 3D,E). Phytohormone quantification data also supported the gene expression data (Fig. 4). Finally, we showed that FL reversed the repression of germination caused by auxin-treatment of soybean seeds, an observation which further demonstrated that IAA indeed inhibits soybean seed germination through promoting ABA biosynthesis (Figs 5, 6, S3 and S4).

A recent study showed that exogenous auxin treatment also inhibited tobacco seed germination^44^. In that investigation, auxin treatment had no significant effect on ABA biosynthesis while GA levels were markedly decreased after exogenous auxin application^44^. However, in the present study, exogenous IAA application promoted ABA biosynthesis while GA_1_ and GA_4_ levels were down-regulated, resulting in decreases in the ratios GA_1_/ABA and GA_4_/ABA (Fig. 4). Further, the gene transcription analysis also supported the phytohormone quantification data (Fig. 3). The differences in the effects of exogenous auxin treatment on ABA or GA biosynthesis during seed germination between this study and the other published research^44^ might be the result of using different crop species in the different studies.

This study revealed that exogenous auxin treatment delayed soybean seed germination. Does this observation have practical applications? In light of the inhibitory effect of auxin during wheat seed germination^28^, we suggest that these findings could be applied to inhibit pre-harvest seed germination in diverse crops, including wheat, rice, maize and soybean. Furthermore, as described above, auxin treatment positively regulated the ABA-biosynthesis pathway and negatively mediated GA biosynthesis through regulation of the transcription of key genes involved in the biosynthetic pathways. In future investigations, the auxin-regulated transcription factors which link the missing gaps between auxins and the ABA- and GA- biosynthesis pathways during the soybean seed germination process, need to be identified and further dissected.

## Materials and Methods

### Plant Materials and Growth Condition

Soybean (*Glycine max* L.) cultivars used in the present study were ND-12 (Nandou-12) and C-103. In detail, both ND-12 (yellow seed coat) and C-103 (black seed coat) are the prevailing soybean cultivars in Southwestern China, and are breeded by Nanchong Academy of Agricultural Sciences, Sichuan Province, P. R. China. These two genotypes are distinct in regard to the genetic background. Both cultivars were grown in Science & Technology Campus, Sichuan Agricultural University, and were harvested at the same time. The elite soybean seeds were chosen for further investigation.

### Seed Germination Phenotypic Analysis

For each repeat, 20 soybean seeds were incubated on 2 layers of medium-speed qualitative filter papers in Petri Dishes (diameter is 9 cm). Then, 11 mL distilled water, 3-indolylacetic acid (IAA, at 1 or 10 μM), α-Naphthalene acetic acid (NAA, at 8 μM), 10 μM 2,4-D, 100 nM fluridone (FL) or 10 μM paclobutrazol (PAC) were add to each plate according to the experiment requirement. Three repeats were performed. The plates were incubated at 25 °C in incubator (Sanyo Versatile Environmental Teat Chamber MLR-350H). Germination was considered when the radicle broke through the seed coat. The germination percentages were counted at different time points. Under dark conditions, the germination rates were recorded by a torch which launches safe green light, according to the previous investigation^45^.

After 48 h of imbibition, the radicles will be cut off, and the length and fresh weight of radicles per 20 seeds were measured. The Image J software was employed to measure the length of radicles. For each germination test, three experimental replications were performed. The average germination percentage ± SE (standard error) of triplicate experiments was calculated. IAA (product number I2886), FL (product number 45511), NAA(product number N0640), 2,4-D (product number N10609) and PAC (product number 33371) were ordered from Sigma-Aldrich Company Ltd., US.

### Investigation of processes of seed germination

In order to understand the microscopic change of soybean seeds during imbibition, the typical imbibed seeds, at different time points (0, 3, 6, 9, 12, 15, 18 hour after sowing), were chosen to take pictures by stereomicroscope (Leica M165 C, Germany). 10 fold magnification was used.

### Gene Expression Analysis

Imbibition seeds were collected at different time points after sowing and frozen in liquid nitrogen quickly. Total RNA preparation, first-strand cDNA synthesis and qRT-PCR assay were performed as our previously described^14^. Quantitative PCR was performed using the SsoFast™ EvaGreen Supermix (Bio-Rad). Each 10 μL reaction comprised 2 μL template, 5 μL SsoFast™ EvaGreen Supermix, 0.5 μL (10 μM) of each primer and 2 μL Dnase-free ddH_2_O. The reactions were set as the following: an initial denaturation step of 95 °C/5 min, 39 cycles of 15 s at 95 °C for denaturation, 30 s at 60 °C for annealing, and 25 s at 72 °C for elongation, and a melting curve analysis was performed at the end of the PCR run over the range 65–95 °C. The transcription pattern of genes involved in ABA/GA biosynthesis and signaling transduction pathways including *GmABA2*, *GmAAO*, *GmCYP707A1*, *GmABI4*, *GmABI5*, *GmRD29-A*, and *GmGA3*, *GmGA3ox2*, *GmGA3ox1*, *GmKAO* and *GmRGL* were investigated. Gene expression was quantified at the logarithmic phase using the expression of the housekeeping *GmACTIN11* as an internal control. Three biological replicates were performed for each experiment. Primer sequences for qRT-PCR are shown in Supplemental Table 1.

### Quantification of ABA in Soybean Seeds

For analysis of ABA content in soybean seeds, the previously protocol we used^14^ were employed in this study. Firstly, the seeds were ground to powder in liquid nitrogen, and 300 mg of seeds powder was homogenized and extracted for 24 hours in methanol containing D6-ABA (OIChemIm Co. Ltd.) as an internal standard. Purification was performed with an Oasis Max solid phase extract cartridge (Waters) and eluted with 5% formic acid in methanol. Subsequently, the elution was dried and reconstituted, and it was then injected into a liquid chromatography–tandem mass spectrometry system consisting of an Acquity ultra performance liquid chromatograph (Acquity UPLC; Waters) and a triple quadruple tandem mass spectrometer (Quattro Premier XE; Waters). Three biological replications were performed.

### Quantification of Endogenous Gibberellins

The endogenous gibberellins were determined according to our method described previously^46^. Soybean seeds (400 mg) were frozen in liquid nitrogen, ground to fine powder, and extracted with 80% (v/v) methanol. GA d_2_ isotope standards were added to plant samples before grinding. The crude extracts were purified by reversed-phase solid-phase extraction, ethyl ether extraction and derivatization. The resulting mixture was injected into capillary electrophoresis-mass spectrometry (CE-MS) for quantitative analysis. Three biological replications were performed.

### Statistical Analysis

The data including germination rates, fresh weight and radicle length of germinated seeds, phytohormones quantification results were analyzed using Student’s T test.

## Electronic supplementary material

Supplemental file
