# Supplementary material for: Exogenous auxin represses soybean seed germination through decreasing the gibberellin/abscisic acid (GA/ABA) ratio
Source: Sci Rep. 2017 Oct 3;7:12620. doi: 10.1038/s41598-017-13093-w (PMC5626727; doi:10.1038/s41598-017-13093-w)
Supplement: Supplementary file 1 — Supplemental file [file 41598_2017_13093_MOESM1_ESM.pdf]

Exogenous auxin represses soybean seed germination through decreasing the gibberellin/abscisic acid (GA/ABA) ratio

Haiwei Shuai[#], Yongjie Meng[#], Xiaofeng Luo, Feng Chen, Wenguan Zhou, Yujia Dai, Ying Qi, Junbo Du, Feng Yang, Jiang Liu, Wenyu Yang[*] and Kai Shu[*]

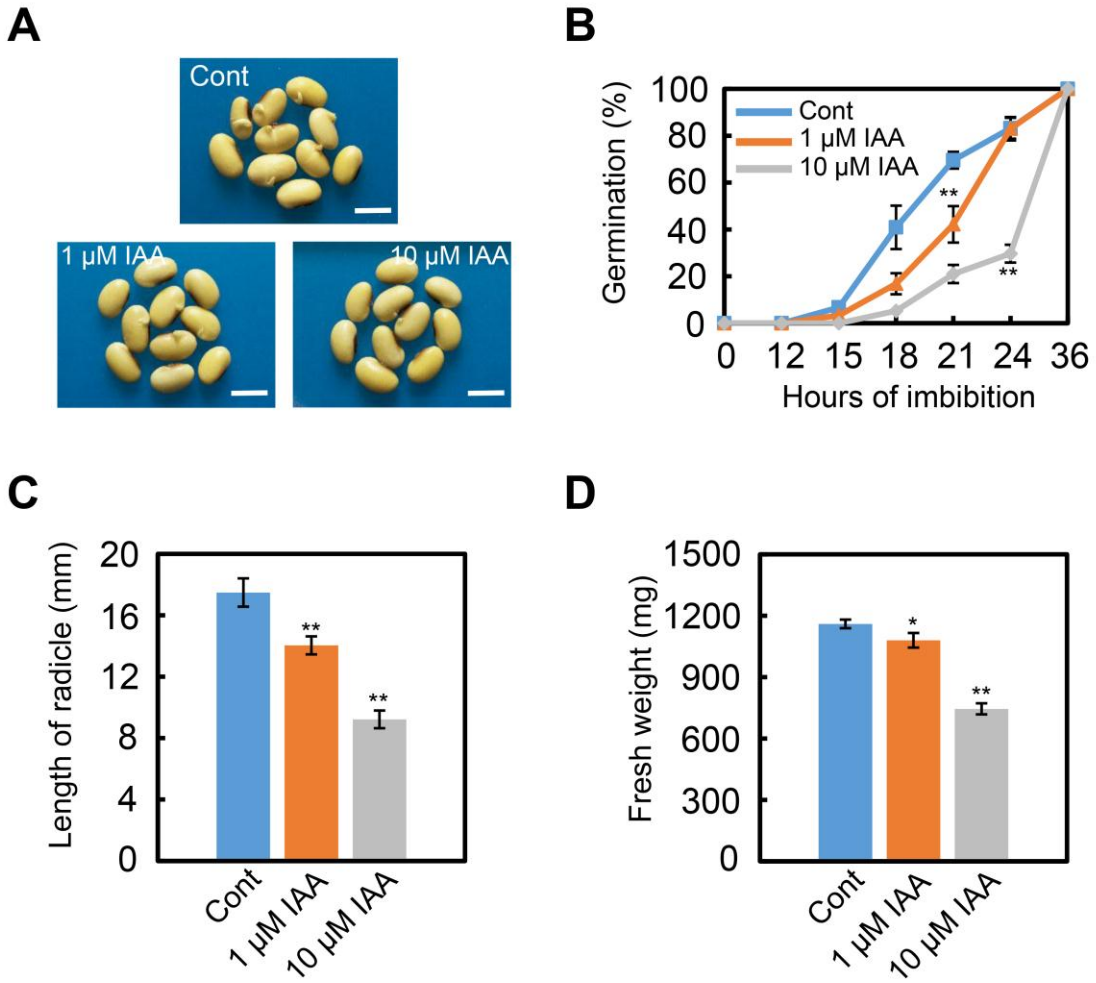

Supplementary Figure S1. IAA inhibits soybean germination in a concentration-dependent manner. Healthy and uniform soybean seeds were treated with different concentrations of IAA. Equivalent amount ultrapure water was added as control (Cont). A, The representative photographs during seed germination (21 hours after sowing) are shown. B, Quantitative analysis of germination rates are shown. C, Length of radicle of germinated soybean seeds after IAA treatment (48 hours after sowing). D, Fresh weight of germinated soybean seeds after IAA treatment (48 hours after sowing). The average percentages of three repeats ± standard error are shown. ** Difference is significant at the 0.01 level, while * Difference is significant at the 0.05 level. Bar in panel A = 10 mm.

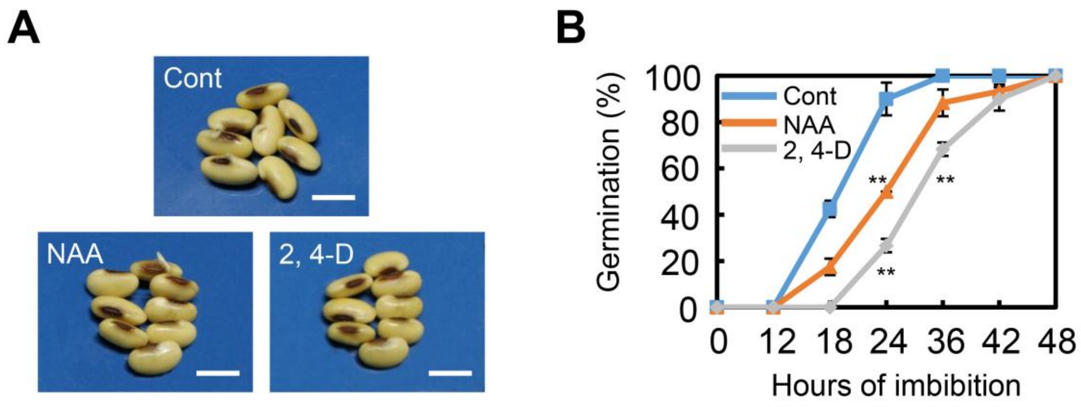

Supplementary Figure S2. Different types of auxin, including NAA and 2, 4-D showed the inhibition effect on soybean seed germination. A, The representative photographs during seed germination (24 h after sowing) are shown. B, Quantitative analysis of germination rates are shown. ** Difference is significant at the 0.01 level. Bar in panel A = 10 mm.

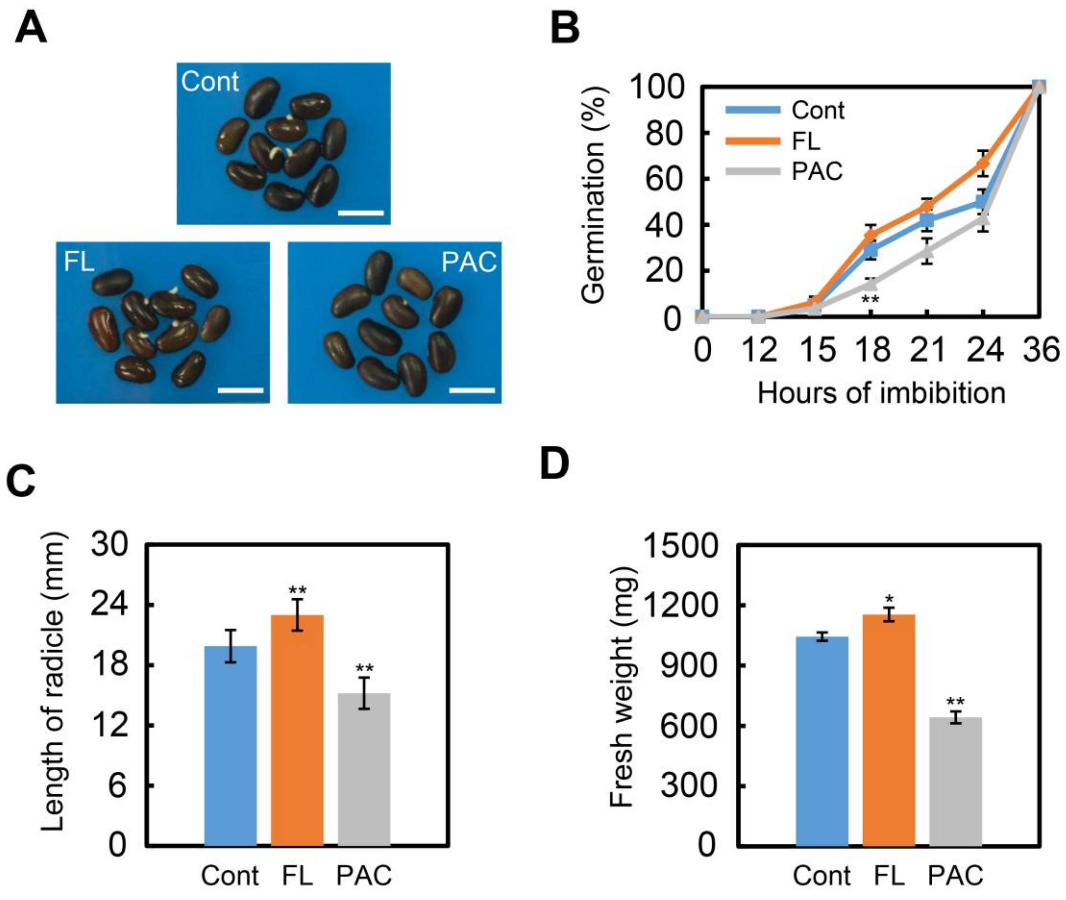

Supplementary Figure S3. The responsiveness of soybean seeds to ABA biogenesis inhibitor FL and GA biogenesis inhibitor PAC. Cultivar C-103 was employed in this experiment. A, The representative photographs during seed germination (21 hour after sowing) are shown. B, Quantitative analysis of germination rates are shown. C, Length of radicle of germinated soybean seeds after FL or PAC treatment (48 hours after sowing). D, Fresh weight of germinated soybean seeds after FL or PAC treatment (48 hours after sowing). ** Difference is significant at the 0.01 level, while * Difference is significant at the 0.05 level. Bar in panel A = 10 mm.

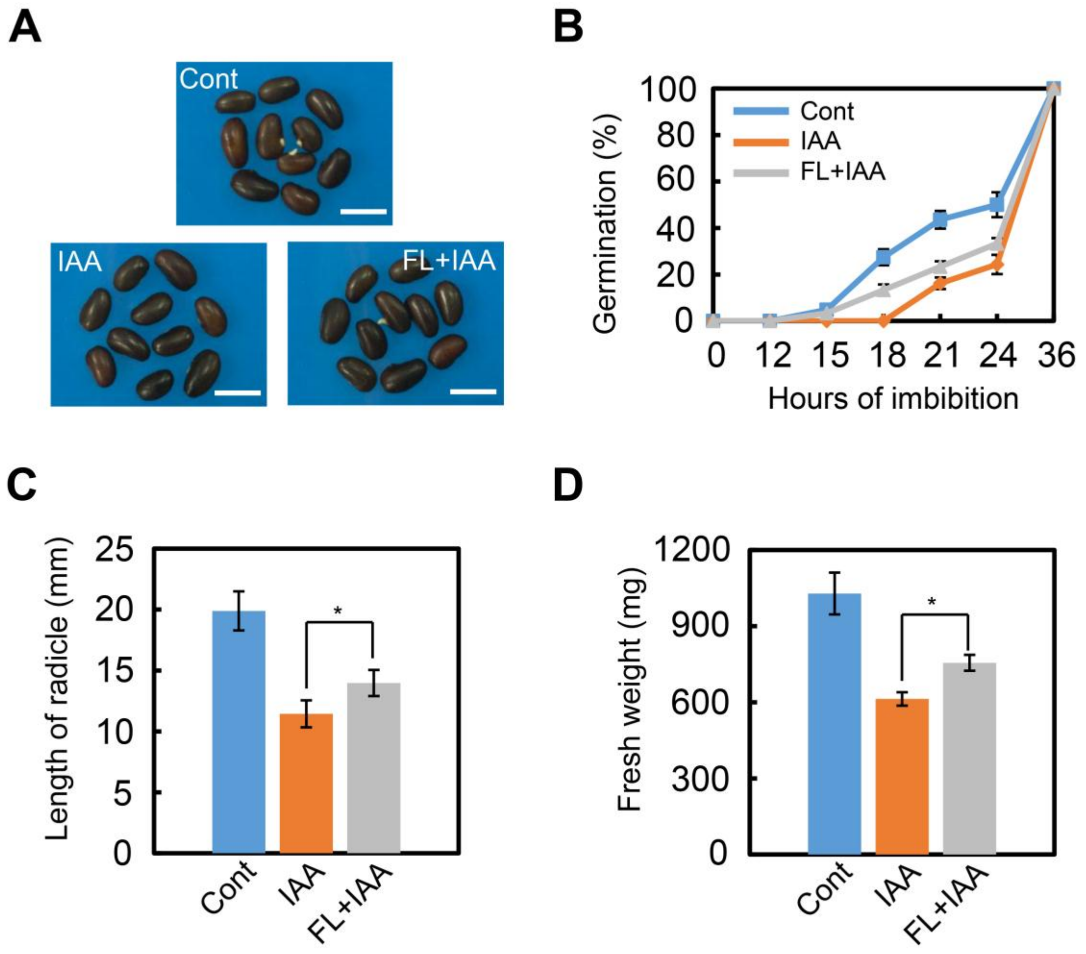

Supplementary Figure S4. FL rescues the delayed-germination phenotype after IAA treatment. Cultivar C-103 was employed in this experiment. Soybean seeds were incubated on two layers of filter paper in Petri dishes at 25 °C and treated with 1 μM IAA and 100 nM FL + 1 μM IAA, respectively. Cont, IAA, IAA + FL three treatments were shown. A, The representative photographs during seed germination (21 hours after sowing) are shown. B, Quantitative analysis of germination rates are shown. C, Length of radicle of germinated soybean seeds after IAA or IAA + FL treatments (48 hours after sowing). D, Fresh weight of germinated soybean seeds after IAA or IAA + FL treatments (48 hours after sowing).

* Difference is significant at the 0.05 level. Bar in panel A = 10 mm.

Supplemental Table 1 Primers for qRT-PCR in this study.

Gene	Forward primer	Reverse primer
GmACT11	ATCTTGACTGAGCGTGGTTATTCC	GCTGGTCCTGGCTGTCTCC
GmAAO	AACTGAAGAAGACACCAACAAG	CTACGCAAGCACCACAAC
GmABA2	CATAGTCAACAATGCTGGAATCTC	ACCTAAGGCACTTGCTACAC
GmABI4	GAATCAACAGCAACAGCAACA	ACCGAAGAAGCATCCATAGC
GmABI5	CGAGTTCCAGCACAGTCT	TGTTCTCTTCAGCGTTCCA
GmRD29A	GGAAGGAAGAGCCAGTGA	AACCAAGAGCCAACAACAC
GmCYP707A	GAGCAGATAGCGGATAATGT	GCACTCTTGTTCCTCAGTAA
GmGA3	AGATTGAACGCACCACACCTT	TCGCAGGAAGAAGAAGAGGATAGA
GmGA3ox	CTCGCATCTCTTCCTTCTTCC	AATCCAACATCAGCCACATCAG
GmGA3ox1	GCCTCCTCCAAGACATTCAA	AGCCATCAACACCGTCAG
GmKAO	TTGGAGGAGGAAGTAGATTGTG	TGCTCTTAGGTTGTTGTAGATGAA
GmRGL	TCCTCGGTCCATCTTCTCT	TTGTTGTTGTTGTTGTTGTTGTC
